# Complete Genome Sequence of a Community-Associated Methicillin-Resistant *Staphylococcus aureus* Hypervirulent Strain, USA300-C2406, Isolated from a Patient with a Lethal Case of Necrotizing Pneumonia

**DOI:** 10.1128/genomeA.00461-17

**Published:** 2017-06-01

**Authors:** Jo-Ann McClure, Kunyan Zhang

**Affiliations:** a Centre for Antimicrobial Resistance, Alberta Health Services/Calgary Laboratory Services/University of Calgary, Calgary, Alberta, Canada; b Department of Pathology & Laboratory Medicine, University of Calgary, Calgary, Alberta, Canada; c Department of Microbiology, Immunology and Infectious Diseases, University of Calgary, Calgary, Alberta, Canada; d Department of Medicine, University of Calgary, Calgary, Alberta, Canada; e The Calvin, Phoebe and Joan Snyder Institute for Chronic Diseases, University of Calgary, Calgary, Alberta, Canada

## Abstract

USA300 is a predominant community-associated methicillin-resistant *Staphylococcus aureus* strain causing significant morbidity and mortality. We present here the full annotated genome of a USA300 hypervirulent clinical strain, USA300-C2406, isolated from a patient with a lethal case of necrotizing pneumonia, to gain a better understanding of USA300 hypervirulence.

## GENOME ANNOUNCEMENT

The community-associated methicillin-resistant *Staphylococcus aureus* (CA-MRSA) pulsed-field gel electrophoresis (PFGE) strain type (pulsotype) USA300 was first reported in the United States in 2000 (1–3). It shortly became a predominant MRSA strain in North America (4–7) and has spread globally (8–12). USA300 has been remarkable not only for its dominance but also for its hypervirulence, causing severe life-threatening diseases, such as sepsis, necrotizing pneumonia, etc. (13–16). The USA300 strain group is composed of a cluster of closely related PFGE patterns, belonging to multilocus sequence type 8 (ST8) of clonal complex 8 (CC8) and carrying staphylococcal cassette chromosome *mec* (SCC*mec*) type IV. In order to elucidate the pathogenicity and virulence of USA300, the whole genomes from many clones of USA300 have been sequenced and compared, and they revealed wide genetic diversity (10, 17–20). A USA300 hypervirulent strain, USA300-C2406, was isolated from a patient with a lethal case of necrotizing pneumonia during our local USA300 outbreak in 2004 (21). It has been used as a virulence control strain in many of our infection models (22–26). We now sequenced the complete genome of USA300-C2406 to give a more complete understanding of its genetic factors and gain a better understanding as to how they relate to its virulence.

The genome of USA300-C2406 was sequenced with Pacific Biosciences (PacBio) RSII sequencing technology, using one single-molecule real-time (SMRT) cell. A total of 103,867 raw reads were generated, covering a total of 1,069,183,823 sequenced bases, with an average read length of 10,293 bp (longest read, 50,205 bp). Contig assembly was done using the HGAP workflow (27–29). The estimated genome coverage was 346×, and the G+C content of the resulting genome was 32.72%. A total of 3 contigs were generated, one of 2,878,854 bp, one of 50,194 bp, and one of 31,144 bp. The largest contig is similar in size to other *S. aureus* chromosomes (~2.8 Mb), while the two smaller fragments show homology to plasmids. Gene annotation was done on the large chromosomal sequence using NCBI’s Prokaryotic Genome Annotation Pipeline. A total of 3,030 genes were identified, of which 2,874 were coding sequences (CDSs), 82 were RNA genes, and 74 were pseudogenes.

Sequence analysis indicates that several virulence factors common in staphylococci were located, including genes for adhesins, like fibrinogen binding (*clfA*, *sdrC*, *sdrD*, and *sdrE*), fibronectin binding (*fnbA* and *fnbB*), elastin binding (*ebpS*), intercellular adhesion (*ica*), and a truncated major histocompatibility complex II (MHCII) analogue (*map*). Genes for enterotoxins *sek* and *seq* were present, as was the immunity evasive gene *scn* and cytotoxin genes *hla*, *hld*, and *hlg*. Genes for exoenzymes, such as the protease *v8*, hyaluronate lyase (*hysA*), and staphylokinase (*sak*) genes, were also found, as was the immunity evasive gene (*chp*). A more complete analysis is under way looking at a broader range of virulence factors and comparing them to those found in less-virulent strains of *S. aureus*, with the goal of elucidating which genes are responsible for the success of USA300.

### Accession number(s).

The chromosomal genome sequence has been deposited in GenBank under the accession number CP019590.
